# Pragmatic trials can address diagnostic controversies: recent lessons from gestational diabetes

**DOI:** 10.1186/s13063-022-06169-0

**Published:** 2022-04-01

**Authors:** Jean Raymond, Hélène Long, Tim Darsaut

**Affiliations:** 1 Department of Radiology, Service of Interventional Neuroradiology, Centre Hospitalier de l’Université de Montréal (CHUM), University of Montreal, Montreal, Quebec H2X 0C1 Canada; 2 Department of Medicine, Division of Endocrinology and Metabolism, Laval Health and Social Services Centres, Laval, Canada; 3 Division of Neurosurgery, Department of Surgery, University of Alberta Hospital, Mackenzie Health Sciences Centre, Edmonton, Alberta Canada

**Keywords:** Diagnostic studies, Pragmatic clinical trials, Research ethics, Gestational diabetes

## Abstract

**Objective:**

The aim of the paper is to discuss how a pragmatic definition could change our conception of diagnosis, using gestational diabetes mellitus (GDM) as an example.

**Study design:**

We review the diagnostic controversy that followed an observational study showing a linear relationship between maternal glycaemia and adverse pregnancy outcomes and the resolution proposed 15 years later by a recent pragmatic trial comparing two screening approaches (one- vs two-step) with different diagnostic thresholds.

**Results:**

The pragmatic trial involved approximately 24,000 women. The one-step screening strategy using lower GDM thresholds diagnosed twice as many women with GDM, but pregnancy outcomes were not different. We examine how the pragmatic approach integrates research into practice and defines the meaning of a diagnosis according to patient outcomes. The approach is ethically and scientifically sound as compared to the previous methodology, where observational research separated from care gave a theoretical definition of GDM that may have misled medical practice for two decades.

**Conclusion:**

Pragmatic research integrated into practice can revolutionize our conception of medical diagnosis in the best medical interest of patients.

## Introduction

Diagnosis is the foundation of clinical decision-making, but contemporary problems of over-diagnosis and over-medicalization have confronted this traditional paradigm. In 1878, Charles Peirce proposed a new, *pragmatic* conception of “meanings”: “Consider what effects, which might conceivably have practical bearings, we conceive the object of our conception to have. Then the whole of our conception of those effects is the whole of our conception of the object” [1].

Applying the pragmatic approach to medical diagnosis, we could say that for a diagnosis to be clinically meaningful, it must have a practical bearing on patient outcomes. Indeed, a recent proposal suggested that diagnosis be replaced by prognosis (or “what is likely to happen”) [2, 3]. This prognostic approach is one step in the pragmatic direction, but it still does not capture all of the practical effects that making a diagnosis has on patients. The impact of prognostic models on patient outcomes, as the impact of making a diagnosis, should also be verified in practice [4]. The effects of making a diagnosis or giving a prognosis on subsequent clinical decisions, treatments, and patient outcomes should be known if we are to practice outcome-based medical care in the best medical interest of patients.

**Fig. 1:**
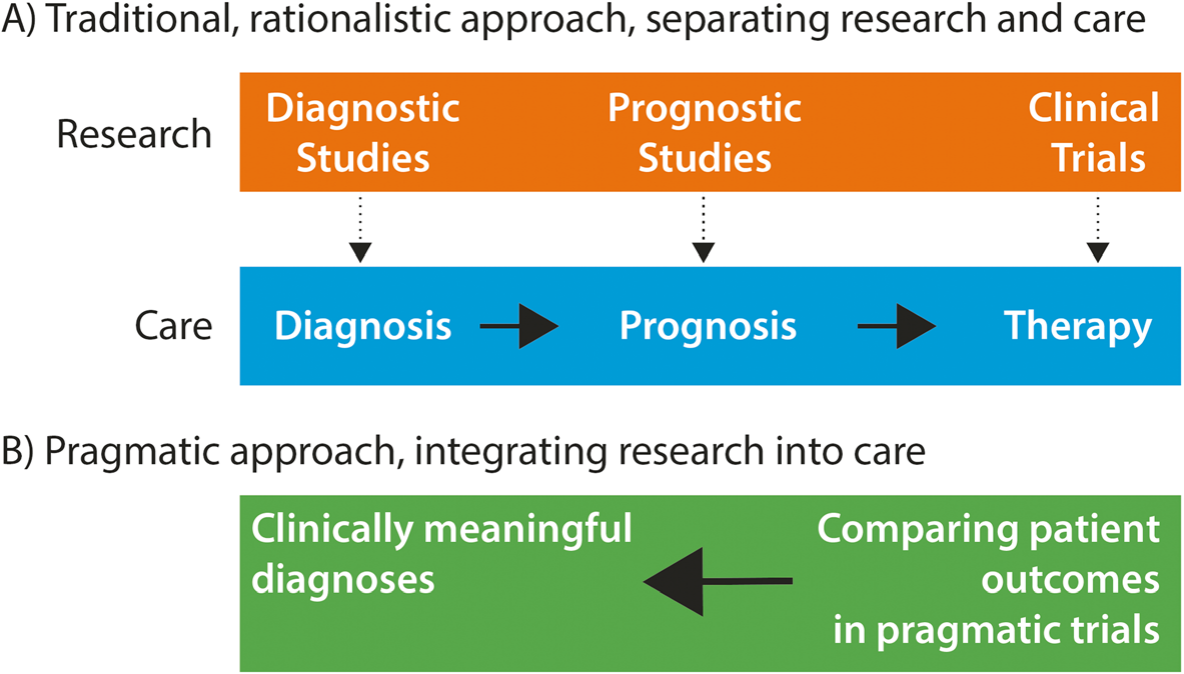
The pragmatic turn. The pragmatic trial approach, to define diagnosis according to clinical outcomes of trials (**B**) is contrasted with the rationalistic approach (**A**)

The pragmatic turn is viewed by many as troublesome, for it seems to reverse the traditional diagnosis-therapy-outcomes order of explanation (Fig. 1). In the present paper, we use the story of gestational diabetes to illustrate how a diagnosis can be defined using the clinical outcomes of a pragmatic trial and how pragmatic trials integrated into practice can revolutionize how some pervasive problems of medicine can be addressed. New diagnoses and new diagnostic criteria should be rigorously assessed for their impact on patient outcomes before being widely recommended.

## Defining gestational diabetes using prognostic observational studies

Gestational diabetes mellitus (GDM) has been defined as glucose intolerance with onset or first recognition during pregnancy [5]. While the label was first used 50 years ago to identify women at risk of overt diabetes *after* pregnancy, GDM has been associated with adverse pregnancy outcomes [6–8]. Unfortunately, because of this vague definition, the diagnosis of GDM has remained controversial and the lack of consensus has perpetuated widely diverging practices.

The Hyperglycemia and Adverse Pregnancy Outcomes (HAPO) study was conducted between 2000 and 2006 to provide internationally acceptable criteria of GDM; 25,000 pregnant women were observed—untreated (except for pre-defined overt hyperglycemia)—to clarify the risks of adverse outcomes associated with various degrees of maternal glucose intolerance less severe than in overt diabetes [9]. Glucose intolerance was assessed with a one-step 75 g oral glucose tolerance test (OGTT) at 24 to 28 weeks, but clinicians were blinded to results. HAPO showed a linear relationship between maternal glucose levels and four primary outcome measures (birth weight above the 90th percentile for gestational age, primary cesarean delivery, clinical neonatal hypoglycemia, and cord-blood serum C-peptide level above the 90th percentile (an index of fetal hyperinsulinemia)). A linear relationship was also observed for secondary outcomes (preeclampsia, preterm delivery, shoulder dystocia/birth injury, hyperbilirubinemia, and admission to intensive neonatal care). There was no inflexion point or observable threshold.

The International Association of Diabetes and Pregnancy Study Group (IADPSG) thought these new research findings should translate into clinical recommendations. The absence of a threshold called for a decision based on expert opinion: in 2010, they redefined GDM as maternal 75 g-OGTT glucose values corresponding to 1.75 times the odd ratios relative to the mean for selected adverse outcomes [8].

The problem is that ready adoption of the new guidelines was expected to double the number of women diagnosed with GDM. Without rigorous evidence of benefit, the new guidelines could unnecessarily medicalize many pregnancies and re-allocate resources away from high risk women [10, 11]. IADPSG experts argued that a new RCT would be costly, time consuming, and unnecessary, because the efficacy of treatment had already been shown by two previous trials [6, 7]. These trials had studied GDM defined by a 2-step screening method using higher diagnostic thresholds, but it was assumed that treatment efficacy would still apply to women identified by the new definition [8].

Numerous (but not all) national diabetic and healthcare associations around the world adopted the 2010 IADPSG criteria. Many “before/after” studies showed that the new criteria substantially increased the number of GDM cases, but the impact on perinatal outcomes remained controversial [12].

## Defining gestational diabetes using the pragmatic trial approach

The controversy centered on two screening methods using different diagnostic cutoffs. The one-step screening approach using HAPO-derived thresholds identified women with mild hyperglycemia as having GDM, but the effects of *identifying and treating* more women with mild GDM were not known.

In March 2021, Hillier and colleagues reported a randomized study involving nearly 24,000 women [13]. This pragmatic trial integrated into practice compared the two screening methods head-to-head; all women could be included because the need for individual informed consent was waived; recruitment difficulties and bureaucratic hurdles were minimized. The rationale was that both screening approaches were associated with minimal risk and were clinically recommended, and clinicians were uncertain regarding which method to use. The results showed that if twice as many women were diagnosed with GDM with the one-step IADPSG approach (16.5% of all pregnancies) compared to the traditional two-step method (8.5%), there were no significant between-group differences in the risks of the primary outcomes relating to perinatal and maternal complications [13].

## Pragmatic lessons from GDM

Our purpose is not to claim that the final word has been established for GDM regarding all health outcomes, for example on long-term outcomes of mothers and their offspring, but rather to examine the wider implications of this story for medical research on diagnosis and care. In our view, the recent pragmatic approach that defined GDM according to *all effects* on neonatal outcomes, including medical interventions, should have been done instead of the observational HAPO study 20 years ago. We want to examine how pragmatism could change our conceptions.

HAPO studied the “natural history of mild GDM” from an outsider’s perspective, without disturbing normal care except for a simple test, keeping practitioners blinded from results for them not to interfere. This approach is in line with the established research-care separation. If observational studies seem at first glance to be less risky than experimenting using randomized trials during pregnancy, in effect HAPO used participants for the sake of theoretical knowledge, because the blinding strategy assured that women could not gain any benefit from participation. To protect subjects, research ethics committees had to consider whether risks were minimized and whether the “generalizable knowledge” to be gained was worth the risk [14]. But the rationalistic research approach may not be the kind of science that is needed to guide medical care. Observational studies can mislead, because they can only show associations. They cannot predict the effects of interventions (good or bad) on patients. But acting on the new knowledge provided by an observational study is hard to resist. IADPSG experts thought they had a duty to translate observational research findings into clinical guidelines. However, they should not have jumped to conclusions, for clinically pertinent GDM diagnostic criteria should require randomized evidence that to act on new glucose thresholds actually improves pregnancy outcomes in practice. RCTs are admittedly difficult endeavors, time-consuming, and costly, but this is a short-sighted view. Assuming the results of Hillier et al. are definitive, the 20-year controversy and the many-thousand pregnancies that have unnecessarily been flagged and treated since HAPO suggest that nothing is more time consuming or costly than to *not* do the RCT [15].

Let us examine an alternative scenario. Imagine that Hillier’s trial had shown that adverse pregnancy outcomes were actually prevented by identifying and treating milder cases of GDM. This scenario would mean that blinding the test results at the time of HAPO deprived all participants from receiving the potential benefits. Study designers would claim that this was not known at the time, but that is exactly the problem: in the presence of uncertainty, the interests of research participants are best protected by balancing risks by using randomized allocation [16]. In our view, the evaluation of HAPO by research ethics committees was doubly erroneous: risks for participants were not minimized (the study did not use randomization to balance risks), and the knowledge to be gained was not worth the risk (since observational research findings ran the risk of misleading medical care for decades).

The pragmatic trial approach may not only apply to many other diagnostic problems involving continuous variables (i.e., hypertension). It may also be the best way to verify the value of diagnostic imaging studies in clinical practice. Some diagnostic questions where there are no treatment implications may not need RCT evidence. But when diagnosis or prognosis affects clinical decisions, we believe it is safest to proceed first with a pragmatic trial.

Medical research dedicated to observations, explanations, and the identification of mechanisms is designed to provide *reasons to act in practice*, but it has long neglected to assess the practical results of these reasons to act on patient outcomes. A pragmatic approach to the design of therapeutic clinical trials was introduced 50 years ago, but only in recent years has it been better understood and promoted [17, 18]. The pragmatic approach may play an equally important role in the definition of meaningful clinical diagnoses. Integrating pragmatic trials into practice can revolutionize medical care in the best interest of patients [19].

## Data Availability

NA
